# Environmental health in crisis—who do we want to be?

**DOI:** 10.1038/s41370-026-00877-4

**Published:** 2026-04-04

**Authors:** Jane E. Clougherty

**Affiliations:** Drexel University Dornsife School of Public Health, Philadelphia, PA USA

I was working on my parent’s back porch in Boston, with my father lying in hospice, days away from passing downstairs, when the email I dreaded appeared: *“… terminated in its entirety effective immediately … no longer consistent with EPA funding priorities.”*

Probably none of us in environmental health could have imagined, in precedented times, that a study on pollution, heat, and child health would be outside of EPA’s scope. A *New York Times* reporter would quote me months later as saying, *“I will never again have to lose my father and my federal funding in the same week. I think I am literally invincible.”*

This particular EPA-funded project used emergency department and hospital data from across New York state to identify community assets associated with lesser impacts of heat or pollution on child health. Research efforts, as we all know, cannot be turned on and off like a light-switch. The expertise needed to work with datasets as large and complex as ours cannot be easily replaced, and the groundwork that goes in to creating such a statewide dataset is immense.

As I’m tremendously fortunate to be tenured at this time, my primary concern at that moment was for my students and staff, who rely entirely on research funding for their salaries. This project, however, also engaged an array of government, clinical, and community partners. They were appropriately disappointed that we would be unable to deliver the results we had promised, which they had relied upon to help inform decisions regarding cost-effective interventions to improve climate preparedness in their communities. Such experiences can ultimately discourage non-academic partners from collaborating with universities at all.

We are living and working through a profoundly challenging moment for environmental health and epidemiology in the U.S. Environmental research has come under direct attack, climate policy is being undermined, and our core principles related to health equity are being threatened. Many of us have lost research funding, and the NIH and EPA funding processes themselves have become politicized.

The structure of our field may be permanently changed. Funding may never return to prior levels, and we may lose much of the next generation of scientists due to lack of funding and discouragement. Life at our home institutions will change, as universities and other institutions now see the precarity of relying on competitive grant funding to offset vast investments in health research infrastructure.

Perhaps most bafflingly, the public and the political tide has turned away from public health—the very sciences that eradicated polio and smallpox, and increased life expectancy drastically in the past century. We are, however, not faultless in this shift; few Americans knew what an epidemiologist was before the Covid pandemic (many still don’t), and we spend far more time quibbling among ourselves about methods and tiny differences in effect size, than in translating the broad sweep of our science to the public and policy-makers. Yes, our scientific credibility and precision absolutely matters—our objectivity, and the reproducibility of our work is the bedrock we stand on. *But what is the right balance of scientific rigor vs. transparency and interpretability? Do small refinements in estimated effect sizes really matter, when decision-makers don’t even care or believe what we have to say??*

*I believe that this is a time for bold, creative thinking to solve the extraordinary challenges facing our field, and to look to what we know about resilience to help get us through*.

I also believe that we have a once-in-a-career opportunity to re-envision our field. If we choose to, we can use this moment to envision a more efficient, effective structure for environmental health going forward. If we’re completely honest with ourselves, things weren’t perfect before: *Who wanted to keep running on the R01 hamster wheel every cycle? What if faculty and senior researchers could actually focus on the science itself, working alongside our trainees, rather than constantly scrambling for funding? What if, instead of competing with each other every NIH cycle, we instead identified collectively the most important scientific questions we still need answered in our field, and pulled together whatever resources we have amongst us, to answer them as efficiently as possible??*

*These can be the times that make us more creative, resilient scientists, if we so choose*. Darwin’s famous quote is often cut short – “It is not the strongest of the species that survives, nor the most intelligent that survives. It is the one that is the most adaptable to change….” He goes on: *“…that lives within the means available and works co-operatively against common threats.”* Following history’s foremost resilience scientist, it behooves us to heed his advice - use the resources we do have as wisely and efficiently as possible, practice cooperation over competition in our research efforts, and start envisioning a more sustainable path forward together. The sooner the better.

